# miR-129 controls axonal regeneration via regulating insulin-like growth factor-1 in peripheral nerve injury

**DOI:** 10.1038/s41419-018-0760-1

**Published:** 2018-06-18

**Authors:** Hui Zhu, Chengbin Xue, Min Yao, Hongkui Wang, Ping Zhang, Tianmei Qian, Songlin Zhou, Shiying Li, Bin Yu, Yongjun Wang, Xiaosong Gu

**Affiliations:** 1 Jiangsu Clinical Medicine Center of Tissue Engineering and Nerve Injury Repair, Research Center of Clinical Medicine, Affiliated Hospital of Nantong University, 20# Xisi Road, Nantong, Jiangsu 226001 P.R. China; 2 Key Laboratory of Neuroregeneration, Ministry of Education and Jiangsu Province, Co-innovation Center of Neuroregeneration, Nantong University, 19# Qixiu Road, Nantong, Jiangsu 226001 P.R. China; 3 State Key Laboratory of Pharmaceutical Biotechnology and MOE Key Laboratory of Model Animal for Disease Study, Model Animal Research Center, Nanjing Biomedical Research Institute., Nanjing University, Nanjing, 210061 P.R. China

## Abstract

The microenvironment of peripheral nerve regeneration consists of multiple neurotrophic factors, adhesion molecules, and extracellular matrix molecules, secreted by unique glial cells in the peripheral nerve system (PNS)-Schwann cell (SCs). Following peripheral nerve injury (PNI), local IGF-1 production is upregulated in SCs and denervated muscle during axonal sprouting and regeneration. Regulation of IGF-1/IGF-1R signaling is considered as a potentially targeted therapy of PNI. We previously identified a group of novel miRNAs in proximal nerve following rat sciatic nerve transection. The present work focused on the role of miR-129 in regulation of IGF-1 signaling after sciatic nerve injury. The temporal change profile of the miR-129 expression was negatively correlated with the IGF-1 expression in proximal nerve stump and dorsal root ganglion (DRG) following sciatic nerve transection. An increased expression of miR-129 inhibited proliferation and migration of SCs, and axonal outgrowth of DRG neurons, which was inversely promoted by silencing of the miR-129 expression. The IGF-1 was identified as one of the multiple target genes of miR-129, which exerted negative regulation of IGF-1 by translational suppression. Moreover, knockdown of IGF-1 attenuated the promoting effects of miR-129 inhibitor on proliferation and migration of SCs, and neurite outgrowth of DRG neurons. Overall, our data indicated that miR-129 own the potential to regulate the proliferation and migration of SCs by targeting IGF-1, providing further insight into the regulatory role of miRNAs in peripheral nerve regeneration. The present work not only provides new insight into miR-129 regulation of peripheral nerve regeneration by robust phenotypic modulation of neural cells, but also opens a novel therapeutic window for PNI by mediating IGF-1 production. Our results may provide further experimental basis for translation of the molecular therapy into the clinic.

## Introduction

Axonal regeneration depends on the intrinsic growth capacity of neurons and the reaction of glial cells, which expresses proteins that inhibit or promote axonal regeneration^1^. Peripheral nerve injury (PNI) initiates a sequential response known as Wallerian degeneration, characterized by axonal degeneration and dedifferentiation of Schwann cells (SCs)^2^, which secrete trophic factors and provide the Büngner’s bands guiding axonal growth^3^. In addition, SCs remove axonal and myelin debris, and secrete cytokines and chemokines that recruit immune cells to the degenerating nerve to further eliminate cell debris^4^, thus speeding up axonal regeneration^5^. Therefore, cellular reactions to stress conditions generated by axonal injury will affect the regenerative capability and therefore functional recovery^1^.

The microenvironment of peripheral nerve regeneration consists of multiple neurotrophic factors, adhesion molecules, and extracellular matrix molecules, secreted by multiple cells including SCs in the peripheral nerve system (PNS). Growth factors play key roles in the regenerative microenvironment following PNI. In neural tissue engineering, different growth factors such as the glial cell-derived neurotrophic factor (GDNF), nerve growth factor (NGF), neurotrophin-3 (NT-3), and the vascular endothelial growth factor (VEGF) are the ones most commonly used in combination or individually^6–8^. The increased expression of insulin-like growth factors (IGFs) in the injured nerve has also been suggested to facilitate axonal regeneration after PNI^9^. The IGF system includes two ligands, their respective receptors, and a family of binding proteins that together regulate a variety of cellular responses^10^. Insulin-like growth factor 1 (IGF-1) is a polypeptide hormone with critical roles in peripheral nerve regeneration. Furthermore, IGF-1 exerts trophic effects on several different cell types in the nervous system, including spinal moto-neurons. Exogenous administration of IGF-1 promotes regeneration of motor axons after axonal lesions in the sciatic nerve and also acts on myelinating SCs and oligodendroglia^11^. A role in the regulation of motor nerve sprouting has been suggested by the observation that IGF-1 increases growth of neurite and branching from embryonic motoneurons and that administration of IGF-1 to adult muscle induces intramuscular nerve branching^12^. IGF-1 signaling pathways have also been associated with age-related neuronal dysfunction and neurodegenerative diseases, such as Parkinson’s and Alzheimer’s disease^13–16^. Rab8a regulates IGF-1 secretion in a GDP-bound form dependent manner^17^.

Owing to the very limited half-life of growth factors in vivo, complicated strategies, including novel carrier materials as microspheres and hydrogels, and other techniques as affinity-based delivery have been applied reluctantly^18^. MicroRNAs, as a potential way of target treatment, are post-transcriptional regulators of gene expression that may be crucial to age- and disease-related changes in growth factor functions^19^. Interestingly, our recent report suggested that the global deregulation of miRNAs in transected sciatic nerve axons and related dorsal root ganglions (DRGs)^20–22^ may influence the axonal regeneration. SCs proliferation and migration were specifically regulated by let-7d/mir-98 through targeting NGF in vitro and in vivo. The downregulation of let-7d stimulated SCs to increase NGF production, which further encouraged axon regrowth^23^. Recently, a functional role of miR-126 has been suggested to be involved in dopamine neuronal cell survival in models of Parkinson’s disease (PD)-associated toxicity^19^. However, the detailed mechanisms of regulation by microRNAs of IGF-1 dysfunctions in sciatic nerve regeneration are still not well understood.

In the present work, our data provided evidence for a novel mechanism of regulating IGF-1/IGF-1R signaling in neurons and SCs by miR-129 and suggest a functional role of this miRNA, broadly, in injured neurons, and the pathogenesis of PNI.

## Materials and methods

### Animals and tissue preparation

Male Sprague–Dawley (SD) rats (220–250 g) were subjected to surgical transection of sciatic nerve as previously described^24^. All animals were then randomly divided into five groups (*n* = 6) according to different time points. The L4-6 DRGs and the 5-mm-long proximal stump segment in five different groups were collected at 0, 1, 4, 7, and 14 days after sciatic nerve transection, respectively. All the experimental procedures involving animals were conducted in accordance with Institutional Animal Care guidelines of Nantong University, China, and approved ethically by the Administration Committee of Experimental Animals, Jiangsu Province, China.

### Tissue immunohistochemistry

The harvested L4-6 DRGs and sciatic nerve segment were fixed in 4% paraformaldehyde, embedded in 5% sucrose, and cut on a cryostat into 12-μm-thick sections, which were immunostained with primary antibodies: mouse anti-neurofilament (NF) 200 (1:400, Sigma), rabbit anti-IGF-1 (1:200, Abcam), and mouse anti-S100 β (1:100), followed by reaction with fluorescently-labeled secondary antibodies (1:400, Invitrogen). Images were taken under fluorescence microscopy (Leica, Germany).

### DRG neuron culture

DRG neurons were dissociated and cultured as described previously^25, 26^ with modifications. Briefly, the L4-L6 DRGs were removed from the adult SD rats, and transferred to Ca^2+^/Mg^2+^-free Hibernate A (BrainBits, Springfield, IL), where the axon roots and dural tissue were manually removed. The DRGs were then transferred to 0.1% collagenase type I (Sigma, St Louis, MO). Following 1.5 h incubation at 37 °C, the DRGs were dissociated in 0.25% trypsin (Gibco) for an additional 15 min at 37 °C, and mechanically triturated through a pipette into the single cell suspension. To remove SCs, a partial purification step was performed by centrifugation at 900 rpm for 5 min on 15% BSA in PBS solution (Sigma). The obtained DRG neurons were cultured on the coated plates in Neurobasal-A and B-27 minus insulin (Gibco) supplemented with penicillin–streptomycin (both 50 U/ml, Gibco). The modulatory effects of IGF-1 on neurite outgrowth were treated with IGF-1 (25 ng/ml; R&D Systems) for 48 h. For the pre-lesion injury assay, we transected the sciatic nerve, waited 4 days, and then cultured adult DRG neurons for 72 h to evaluate their regeneration capacity.

### Primary culture of SCs and cell transfection

SCs were isolated from sciatic nerves of 1-day-old SD rats and treated to remove the fibroblasts using anti-Thy 1.1 antibody (Sigma, St Louis, MO) and rabbit complement (Invitrogen, Carlsbad, CA) as previously described^27^. The final cell preparation consisted of 99% SCs, as determined by immunostaining with S100 β, a specific SC marker. Primary culture of SCs was maintained in Dulbecco’s modified Eagle’s medium (DMEM) containing 10% fetal bovine serum (complete medium) at 37 °C under humidified 5% CO_2_. The cell culture was passaged no more than three times before the following tests. Primary cultured SCs were transfected with miRNA mimic, miRNA inhibitor, or siRNAs (Ribobio, Guangzhou, China), respectively, using Lipofectamine RNAiMAX transfection reagent (Invitrogen), according to the manufacturer’s instructions. A non-related, scrambled miRNA was used as a control, such as miRNA mimic control (NC) and inhibitor control (anti-NC). Mutated miR-129 mimic was used to confirm the specific effect by miR-129.

### Luciferase reporter assay

The whole 3′-UTR sequences of IGF-1 (NM_001082479) were amplified from the genomic DNA with appropriate primers, and sub-cloned into the pmiR-RB-REPORT^TM^ vector (Ribobio, Guangzhou, China) with the XhoI and NotI sites downstream of the hRluc reporter gene. This vector is based on dual-luciferase technology, with firefly Renilla luciferase (hRluc) used as the primary reporter to monitor mRNA regulation and luciferase (hluc) acting as a control reporter for normalization. Mutant 3′-UTR reporter plasmid was constructed with a QuikChange kit (Stratagene). Primers used to generate wild-type and mutant IGF-1 3′-UTR were as follows: IGF-1 3′-UTR wild-type: AATTCTAGGCGATCGCTCGAGGAGGAGCCTCCCGAGGAACAG, ATTTTATTGCGGCCAGCGGCCGCCCTAATTTTGTCCTTTTGGGCTC; IGF-1 3′-UTR mutant 1: GCAAGGTGCAAAGCTTTTTTTTTTTGTTTTTGAAAAACTTTTTTTTTTTTTTTTTTTTAACAAACACTCCTAAAGACAATGTCGGAATGTTTACTT, AAGTAAACATTCCGACATTGTCTTTAGGAGTGTTTGTTAAAAAAAAAAAAAAAAAAAAGTTTTTCAAAAACAAAAAAAAAAAGCTTTGCACCTTGC; IGF-1′-UTR mutant 2: CAGGACCACTTTTGCAAGGTGCAAAGCTTTTTTAAAAACTTTTTGTTTTTGTTTTTTTTTTTTTTTTTTTTAACAAACACTCCTAAAGACAAT, ATTGTCTTTAGGAGTGTTTGTTAAAAAAAAAAAAAAAAAAAACAAAAACAAAAAGTTTTTAAAAAAGCTTTGCACCTTGCAAAAGTGGTCCTG. The sequences of wild-type and mutant 3′-UTR were confirmed by sequencing. For reporter assays, HEK 293T cells were co-transfected with wild-type (mutant) reporter plasmid and miRNA mimic or scrambled control (NC) by Lipofectamine 2000 (Invitrogen). Firefly and Renilla luciferase activities were measured in cell lysates using the Dual-Luciferase Reporter Assay system. After 36 h incubation, the activity of firefly and Renilla luciferases was measured (Promega). Activity was reported by normalizing Renilla to firefly luciferase activity. Luciferase experiments were repeated three independent times in triplicate. Data are presented as means ± SEM.

### Quantitative real-time RT-PCR (qRT-PCR)

Total RNA was isolated from tissues and cells using Trizol reagent (Invitrogen), and cDNA was prepared from total RNA using a Prime-Script RT reagent Kit (TaKaRa, Dalian, China) according to the manufacturer’s instructions. qRT-PCR was performed with SYBR Premix Ex Taq (TaKaRa) on an ABI system (Applied Biosystems, Foster City, CA) according to standard protocols. The relative expression of miR-129 was quantified with stem-loop RT primers (Ribobio) according to manufacturer’s instructions and normalized against the U6 level. The sequences of IGF-1 primers are as follows: GACCAAGGGGCTTTTAC, TCAGATCACAGCTCCGG. All reactions were run three independent times in triplicate. The relative expression was calculated using the comparative 2^−ΔΔCt^ method.

### Western blot analysis

Protein extracts were prepared from nerve tissues. Equal amounts of isolated protein were separated on 10% SDS-PAGE and transferred to PVDF membranes (Millipore), which were blocked with 5% nonfat milk in Tris–HCl buffered saline (TBS) at room temperature and probed with primary antibodies against IGF-1 (Proteintech) and IGF-1 Receptor (Abcam), respectively, and then reacted with an HRP-conjugated species-specific secondary antibody, followed by an enhanced chemiluminescence assay (Pierce, Rockford, IL).

### ELISA

Primary SCs were transfected with IGF-1 siRNA and negative control, miR-129 mimic and control, miR-129 inhibitor and control, respectively. Afterwards, the medium was replaced with FBS-free DMEM for additional incubation. The medium was then taken out and filtered through a 0.22 μm filter (Millipore) to furnish the supernatant. The protein level of IGF-1 in the medium was measured with the IGF-1 ELISA Kit (R&D Systems) according to the manufacturer’s instructions. The data were measured and averaged from three independent cultures, each comprising triplicate wells.

### Cell proliferation assay

SCs were plated at a density of 1 × 10^5^ cells/ml onto poly-L-lysine-coated 96-well plates. At the indicated time points after cell transfection, 50 mM EdU was applied to the cell culture which was then incubated for an additional 24 h. The cells were fixed with 4% paraformaldehyde in PBS for 30 min. After labeling, the cells were analyzed using a Cell-Light EdU DNA Cell Proliferation Kit (Ribobio) according to the manufacturer’s protocol. SC proliferation was expressed as the ratio of EdU-positive cells to total cells, which was determined using images of randomly selected fields obtained on a DMR fluorescence microscope (Leica Microsystems). Assays were performed three times using triplicate wells.

### Cell migration assay

The migration ability of SCs was examined using 6.5 mm transwell chambers with 8 μm pores (Costar, Cambridge, MA). The bottom surface of each membrane was coated with 10 μg/ml fibronectin. 100 μl Primary SCs (3 × 10^5^ cells/ml) were resuspended in DMEM and transferred to the top chambers of each transwell to allow their migration in a humidified 5% CO_2_ incubator at 37 °C with 500 μl complete medium being pipetted into the lower chambers. The upper surface of each membrane was cleaned with a cotton swab at the indicated time point. Cells adhering to the bottom surface of each membrane were stained with 0.1% crystal violet and then counted under a DMR inverted microscope (Leica Microsystems). Assays were performed three times using triplicate wells.

### Co-culture of DRG neurons and SCs

Primary SCs were transfected with miR-129 mimic and control, miR-129 inhibitor and control, respectively, by the above-described protocols, and then co-cultured with rat DRG neurons, which have been transfected with IGF-1R siRNA and control previously. Neurons were re-suspended and re-plated to allow neurites to regrow. After 48 h co-culture of SCs with DRGs in transwell chambers (1 μm pores, Costar, Cambridge, MA), DRG neurons were isolated, and fixed with 4% paraformaldehyde to undergo immunocytochemistry with anti-β-Tubulin III antibody (Sigma) to observe axon outgrowth. For parallel experiment on SC migration, DRG neurons were transfected with miR-129 mimic and control, miR-129 inhibitor and control, respectively. Then SCs with IGF-1R knocking down previously were transferred to the top chambers of each transwell, and were co-cultured with transfected DRG neurons.

### In vivo experiments

Adult male SD rats were anesthetized before the sciatic nerve was exposed through an incision on the left hind limb and transected to create a gap. A silicone tube (i.d. 1.0 mm) was implanted to bridge the nerve gap with the proximal nerve stump anastomosed to the tube at the junction. The rats were randomly divided into four groups (*n* = 6 each) to receive injection of a mixture of Matrigel (BD Biosciences, Billerica, MA) with purified IGF-1 protein (100 μg/ml; R&D Systems) or PBS vehicle only, and miR-129 inhibitor (antagomir, Ribobio) or corresponding control (Ribobio) both at a volume ratio of 1:1, respectively. The injection was performed from the opposite opening of the silicone tube into the tube lumen using a precooled micropipette, followed by anastomosis of the tube to the distal nerve stump at the junction. The injection was done as slowly as possible to prevent the formation of air bubbles. Afterwards, the surgical incision was closed in a routine fashion, and animals were housed in large cages. At 10 days after surgery, rats were killed and the silicone tube, together with regenerated nerves, was harvested for cutting into sections, which were subjected to immunohistochemistry anti-NF200 and anti-S100β (both from Sigma), respectively. To assess the axons regeneration or the migration of SCs within the nerve gap, the edge of both proximal and distal nerve stump was labeled. Axons or SCs were identified as NF200- or S100β-positive cells, respectively. Then the length of regenerative axons from the proximal or SCs migration from the distal nerve stump was measured.

### Statistical analysis

Data are presented as means ± SEM. The Student’s *t*-test or ANOVA was used for statistical analyses by the aid of SPSS Statistics 22.0 software package (IBM, Chicago, IL). Statistical significance was accepted at *p* value < 0.05.

## Results

### Temporal changes of IGF-1 expression following sciatic nerve injury

**Fig. 1:**
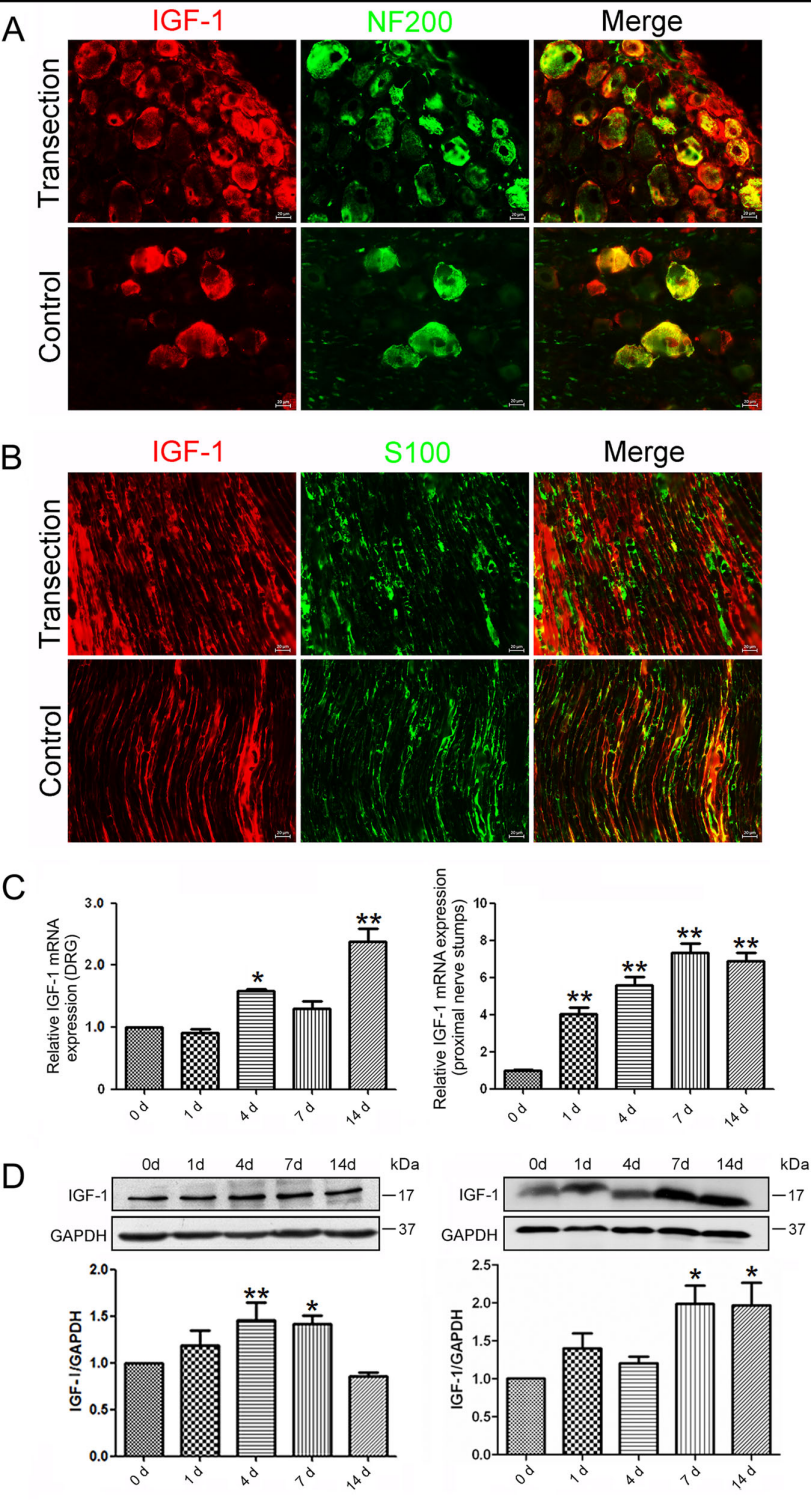
Temporal changes of IGF-1 after sciatic nerve transection. Localization of IGF-1 in the DRGs (**a**) and proximal nerve stumps (**b**) by immunohistochemistry at 0 and 7 days following sciatic nerve injury. SCs and DRGs were labeled with S100 and NF200, respectively. Scale bar: 20 μm. The expression change of IGF-1 at mRNA (**c**) and protein levels (**d**) in DRGs and proximal nerve segment following sciatic nerve transection, respectively. *p < 0.05 and ** *p* < 0.01 versus control (0 h). GAPDH served as internal reference

IGF-1 is important for the PNS response to injury and beneficial to axonal regeneration^12^. Double immunofluorescence staining for S100β (a SCs marker) and NF200 (a neuron marker) were used to determine the cellular localization of IGF-1. The results showed that IGF-1 expressed in DRG neurons and SCs of the sciatic nerve (Fig. 1a,b). After sciatic nerve transection, IGF-1 was showed up-regulated in DRGs and SCs of the proximal nerve segment at 7 days following sciatic nerve injury.

The qPCR analysis showed mRNA expression of IGF-1 in the proximal nerve segment after sciatic nerve transection was significantly increased at 1, 4, 7, and 14 days following sciatic nerve injury as compared to that at 0 h (control) and a peak value occurred at 7 days after sciatic nerve transection (Fig. 1c).

Western blot analysis showed that protein expression of IGF-1 was not significantly increased at 1 day after sciatic nerve transection, but was extensively increased at 7 and 14 days compared to that at 0 h, with a peak value at 7 days (Fig. 1d). The protein expression profile of IGF-1 was not exactly parallel to the mRNA expression profile of IGF-1, suggesting that the expression IGF-1 might be regulated at the post-transcriptional level. The expression of IGF-1 at mRNA and protein levels were also increased at 4, 7, and 14 days following sciatic nerve injury compared to that at 0 h (control) in DRGs, which was a less dramatic change than that in the proximal nerve segment. These suggested that the injury-induced IGF-1 secretion is mainly caused by SCs and DRG neurons at the nerve injury site, and locally promoted axon regeneration.

### miR-129 negatively regulated IGF-1 by directly targeting its 3′-UTR

**Fig. 2:**
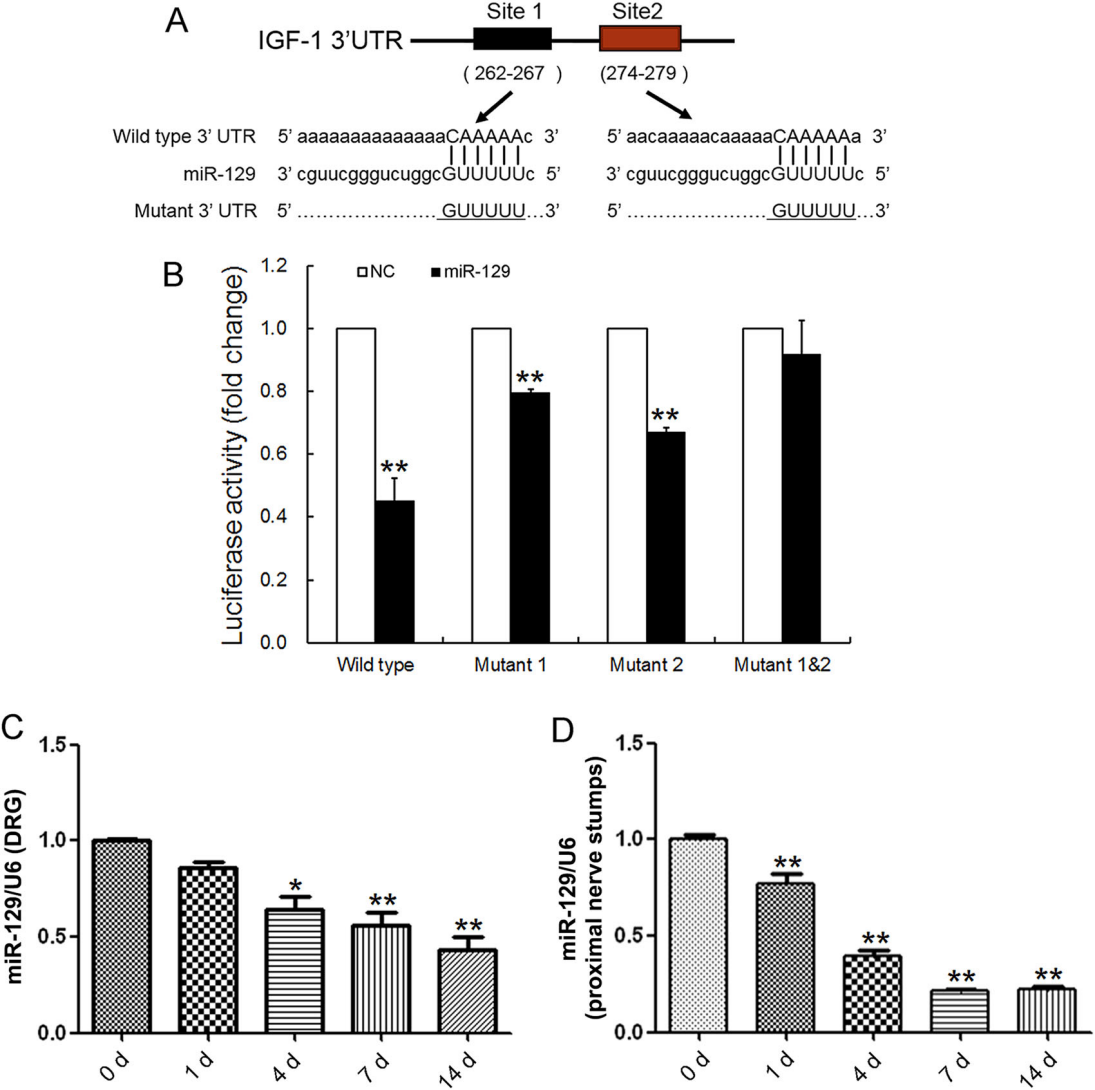
miR-129 was an upstream regulator for IGF-1. **a** Sketches of the construction of wild-type or mutant IGF-1 3′-UTR reporter plasmid for miR-129. **b** The relative luciferase activity was analyzed after the wild-type or mutant IGF-1 3′-UTR reporter plasmid co-transfected into HEK 293T cells with miR-129 mimic (miR-129) or mimic control (NC). **p < 0.01 versus control. Expression of miR-129 in DRGs (**c**) and proximal nerve stumps (**d**) following sciatic nerve transection. * *p* < 0.05 and ** *p* < 0.01 versus control (0 h post nerve injury). U6 served as internal reference

Most miRNAs perform their biological functions by directly binding to the 3′-UTR of their target genes. To screen potential miRNAs that regulate IGF-1 expression, we used bioinformatic database including TargetScan and miRanda, together with our previous data^21, 22^ from mRNA and miRNA microarray assays in DRG tissue and the proximal nerve stump following sciatic nerve injury. It indicated that four miRNAs, including miR-129, miR-18a, miR-206, and miR-340-5p, might regulate the important nerve regeneration-associated gene IGF-1. To verify which one/ones of them were exact regulators, the wild-type and mutant 3′-UTR of IGF-1 sub-cloned into the luciferase reporter vector including single target site mutant (mut 1 and mut 2) and double target site mutant (mut 1&2) were constructed and inserted into the reporter plasmid (Fig. 2a). We noted that miR-129 significantly reduced the luciferase activity of the 3′-UTR of IGF-1 (Fig. 2b). The reduction in the relative luciferase activity was less dramatic when cells co-transfected with single target site mutant (mut 1 or mut 2), while this decrease was abrogated with double target site mutants (mut 1&2). The results suggested that miR-129 directly inhibited IGF-1 expression by binding to a defined target sequence. To verify the correlation between miR-129 and IGF-1 expressions, the expression profiles of miR-129 in DRGs and the proximal nerve segment at 0 h, 1, 4, 7, and 14 days following nerve injury were investigated (Fig. 2c,d). The miR-129 expression in the proximal nerve segment was significantly decreased at 1, 4, 7, and 14 days following nerve injury as compared to that at 0 h (control). Similarly, the decrease of miR-129 was less dramatic in DRGs than that in the proximal nerve segment. Interestingly, the comparison between Figs. 1 and 2 suggested that the temporal expression profile of miR-129 was negatively correlated with that of IGF-1, suggesting miR-129 negatively regulated the IGF-1 expression.

### miR-129/IGF-1 axis regulates neurite outgrowth from DRG neurons

**Fig. 3:**
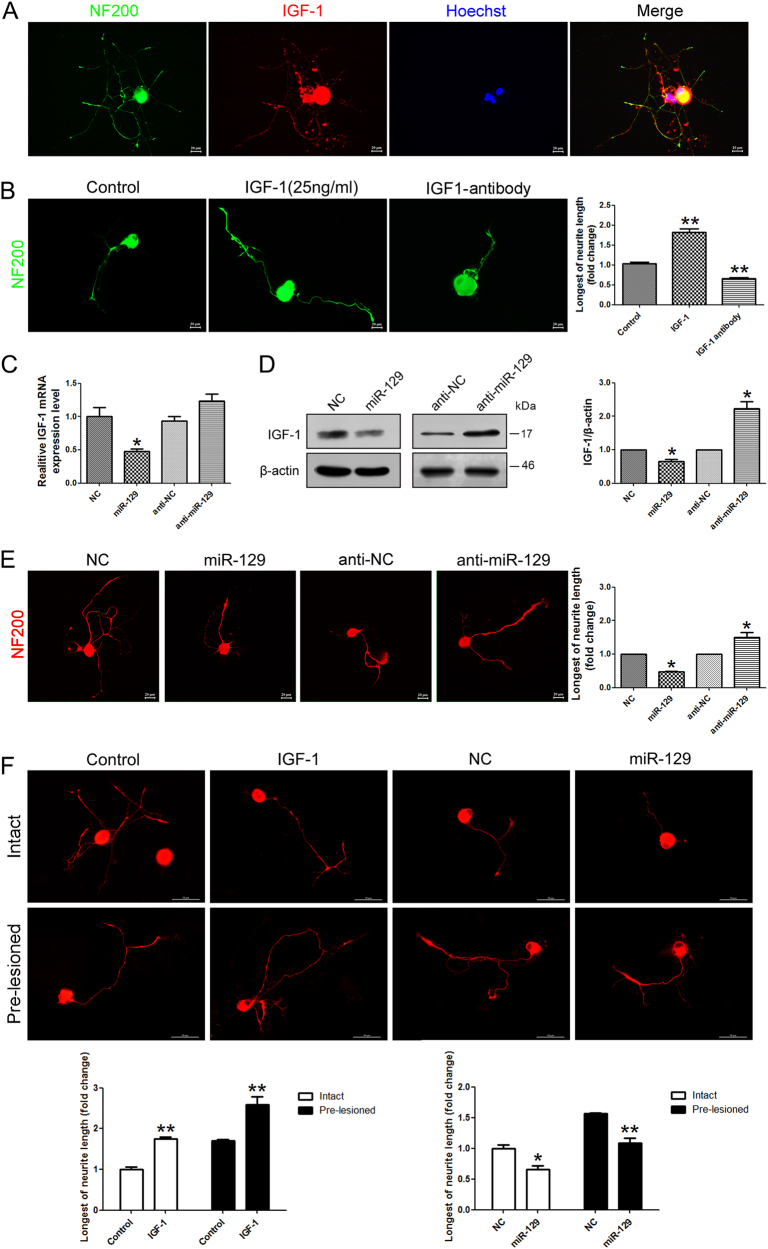
miR-129/IGF-1 axis regulates neurite outgrowth from DRG neurons. **a** Co-localization of IGF-1 and NF-200 in DRG neurons. **b** Neurons treated with IGF-1 (25 ng/ml) for 48 h showed increased neurite outgrowth while IGF-1-neutralizing antibody reduced the effect. Scale bar: 20 μm. The longest neurite outgrowth from DRG neurons was observed and compared with control. The expression change of IGF-1 at mRNA (**c**) and protein levels (**d**) in DRG neurons transfected with miR-129 mimic (miR-129) and miR-129 inhibitor (anti-miR-129). β-actin was used as an internal control. **e** Transfection of miR-129 mimic or inhibitor in DRG neurons, respectively. **f** A prior lesion of the sciatic nerve promoted axon regeneration in intact DRG neurons, meanwhile the IGF-1 treatment enhanced the axon regrowth. The regrowth effect was abolished in miR-129 mimic transfected DRG neurons. Scale bar: 20 μm, *p < 0.05 and **p < 0.01 versus respective controls. NC (mimic control), anti-NC (inhibitor control)

It is well known that IGF-1 exerts autocrine and paracrine effect in neurite outgrowth, which is an important aspect of neuronal development^10^. IGF-1 is a neurotrophic factor for neurite outgrowth. We firstly explored the role of miR-129/IGF-1 axis in DRG neurons. Immunofluorescence staining showed co-localization of IGF-1 and NF-200 in DRG neurons, suggesting that DRG neurons express IGF-1 (Fig. 3a). Exogenous addition of IGF-1 to cell culture medium could significantly increase the neurite outgrowth of DRG neurons, whereas IGF-1-neutralizing antibody reduced this effect (Fig. 3b). To investigate the effects of miR-129 on the expression of endogenous IGF-1 in DRG neurons, qRT-PCR (Fig. 3c) and western blot (Fig. 3d) were applied. It showed the expression of endogenous IGF-1 mRNA and protein decreased in primary DRG neurons transfected with miR-129 mimic, while the IGF-1 expression was upregulated with miR-129 inhibitor. It indicated that miR-129 caused the mRNA degradation of IGF-1. We also observed that transfection of miR-129 mimic (miR-129) significantly impaired the outgrowth ability of DRG neurons when compared to mimic control (NC) cells followed by neurite outgrowth assay (Fig. 3e). These data suggested that miR-129/IGF-1 axis regulated neurite outgrowth from DRG neurons. For further evaluation of the miR-129/IGF-1 axis in the pre-lesion response, we assessed axon regrowth in pre-lesioned DRG neurons. Prior lesion of the sciatic nerve promoted axonal regeneration in intact DRG neurons, meanwhile the IGF-1 treatment enhanced the axonal regrowth (Fig. 3f). However, the accelerated growth of axon was blocked in miR-129 mimic transfected neurons. Our data showed that miR-129/IGF-1 axis also regulates the injury-induced acceleration in injured neurons.

### miR-129 inhibited IGF-1 secretion of SCs

**Fig. 4:**
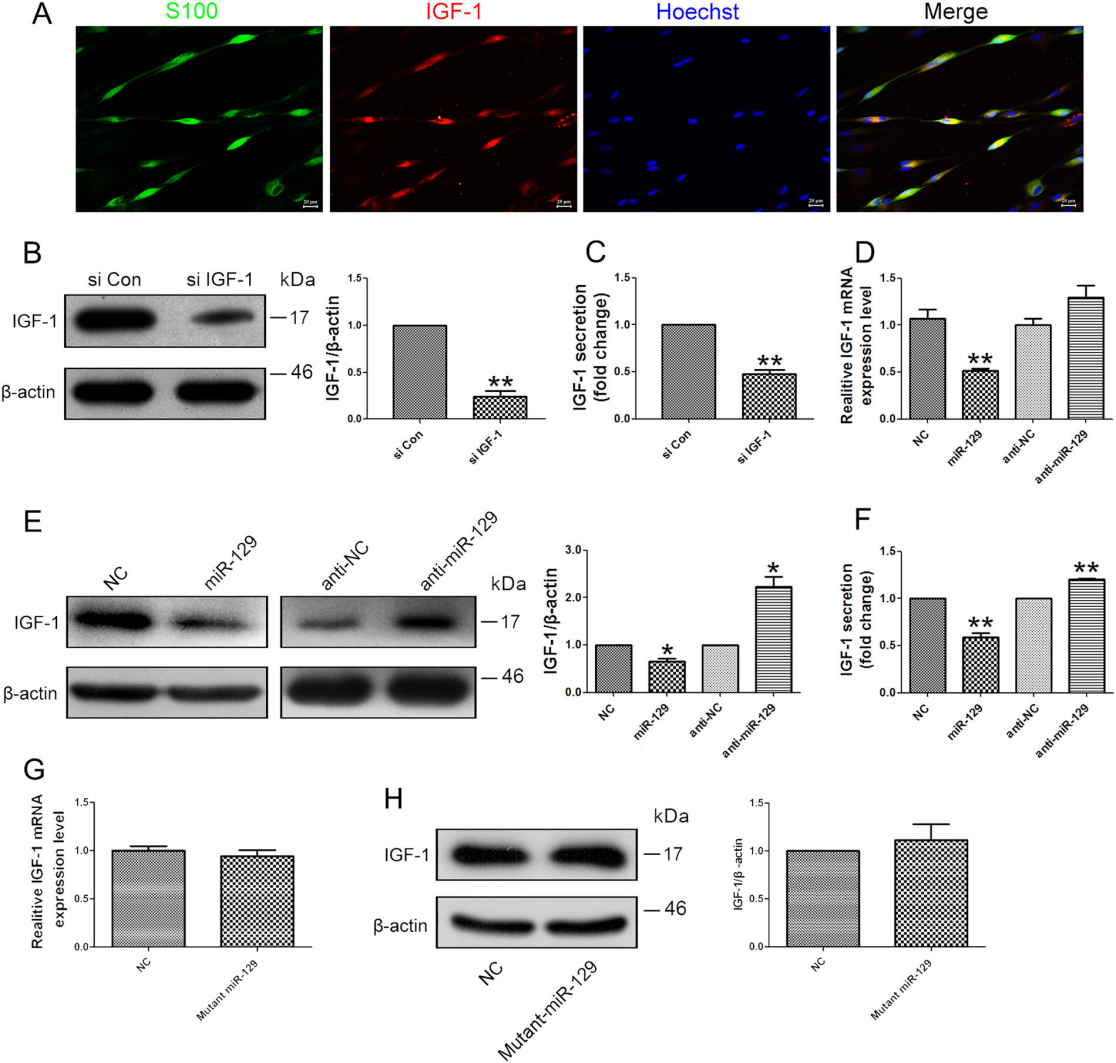
miR-129 inhibited IGF-1 secretion from SCs. **a** Co-localization of IGF-1 and S100 in SCs. The mRNA (**b**) and protein (**c**) expression of IGF-1 as well as the IGF-1 secretion in primary SCs transfected with IGF siRNA (si IGF-1) was significantly decreased as compared to that in SCs transfected with siRNA control (si Con). The expression change of IGF-1 at mRNA (**d**) and protein levels (**e**) in SCs transfected with miR-129 mimic (miR-129) and miR-129 inhibitor (anti-miR-129). β-actin was used as an internal control. **f** IGF-1 secretion was reduced from SCs transfected with miR-129 mimic (miR-129) but was increased from SCs transfected with miR-129 inhibitor (anti-miR-129). **g** The expression of IGF-1 was not significantly affected by mutant miR-129 mimic both on mRNA level and protein level (h). *p < 0.05 and **p < 0.01 versus respective controls. NC (mimic control), anti-NC (inhibitor control), Mutant miR-129 (mutated miR-129 mimic)

Following PNI, SCs secret IGF-1 at the injury site to promote nerve regeneration. In the current study, ELISA analysis was performed to investigate the effects of miR-129 on IGF-1 production from SCs. Firstly, we verified the co-localization of IGF-1 and S100β in SCs by immunofluorescence staining (Fig. 4a). After stable knockdown of IGF-1 in primary SCs (Fig. 4b), IGF-1 secretion was significantly reduced (Fig. 4c). To identify the effect of miR-129 on the expression of IGF-1 in SCs, miR-129 mimic or inhibitor were respectively transfected into SCs, and the mRNA and protein expressions of IGF-1 were respectively testified. The qRT-PCR (Fig. 4d) and western blot analysis (Fig. 4e) showed the mRNA expressions of IGF-1 were significantly suppressed by over-expression of miR-129, whereas were significantly enhanced by silencing of miR-129. Moreover, miR-129 over-expression induced decrease in IGF-1 secretion from SCs, but miR-129 inhibitor failed to show the same effect (Fig. 4f). These indicate the effects of IGF-1 knockdown on SCs are similar to the effects of miR-129 mimics. The results of mutated miR-129 mimic further confirmed the regulation of IGF-1 by miR-129 (Fig. 4g, h).

### miR-129 suppressed SCs proliferation and migration

**Fig. 5:**
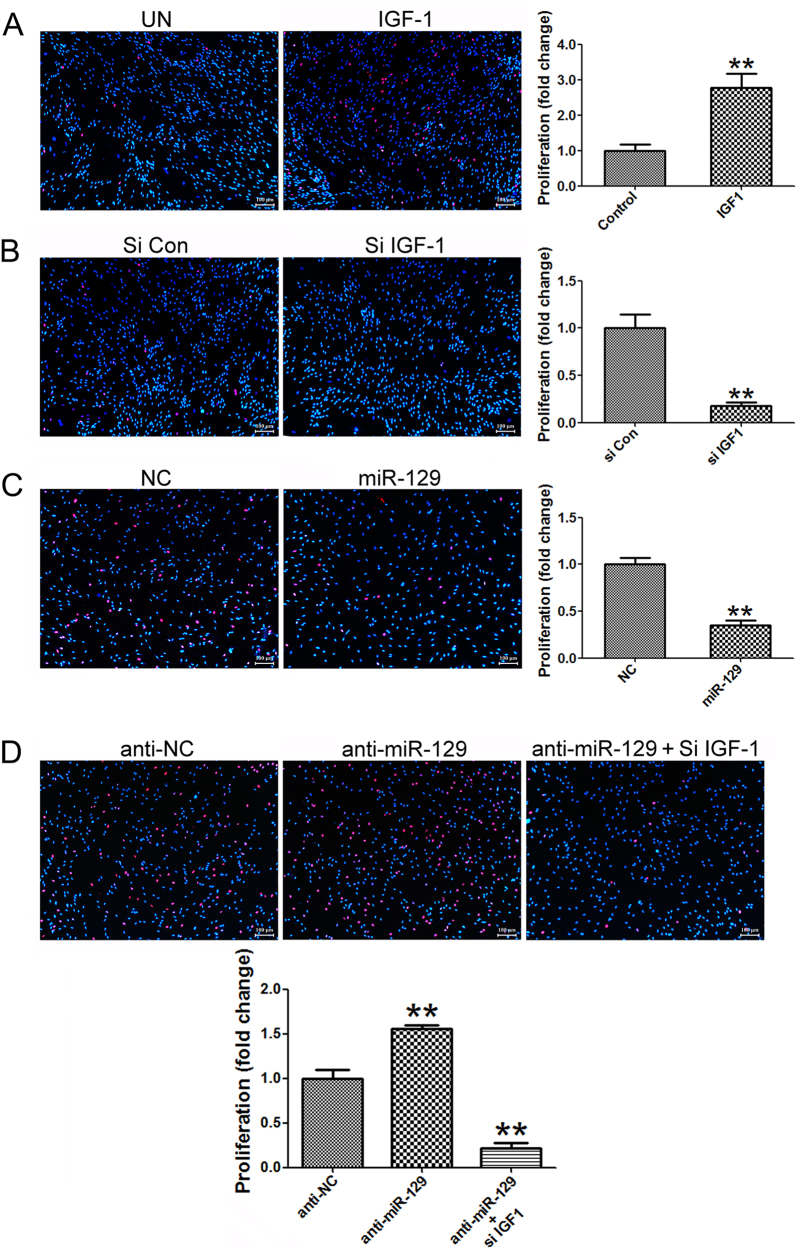
miR-129/IGF-1 axis regulates proliferation of SCs. **a** IGF-1 promoted proliferation of SCs by IGF-1 treatment. **b** Knocking down of IGF-1 by siRNA (si IGF-1) reduced this effect. **c** The proliferation rate of SCs transfected with miR-129 was significantly decreased while the proliferation rate of SCs transfected with miR-129 inhibitor was significantly increased compared with that of control (**d**), but was then rescued by co-transfection with miR-129 inhibitor plus IGF-1 siRNA (anti-miR-129+ si IGF-1). **p < 0.01 versus respective controls. NC (mimic control), anti-NC (inhibitor control)

As noted above, IGF-1 is secreted partially by SCs following nerve injury and is noted as a crucial factor in SCs biology. Exogenous addition of IGF-1 promoted SCs proliferation (Fig. 5a), while IGF-1 knocking down inhibited SCs proliferation (Fig. 5b). EdU incorporation results showed that over-expression of miR-129 reduced the proliferation rate of SCs to less than 50% of the control value while silencing of miR-129 increased the proliferation rate of SCs to nearly 1.5 folds compared to the control value, suggesting that miR-129 could suppress SC proliferation (Fig. 5c). Conversely, miR-129 inhibitor induced increase in SCs proliferation was significantly abrogated by IGF-1 knockdown after SCs were co-transfected with miR-129 inhibitor and IGF-1 siRNA (Fig. 5d).

**Fig. 6:**
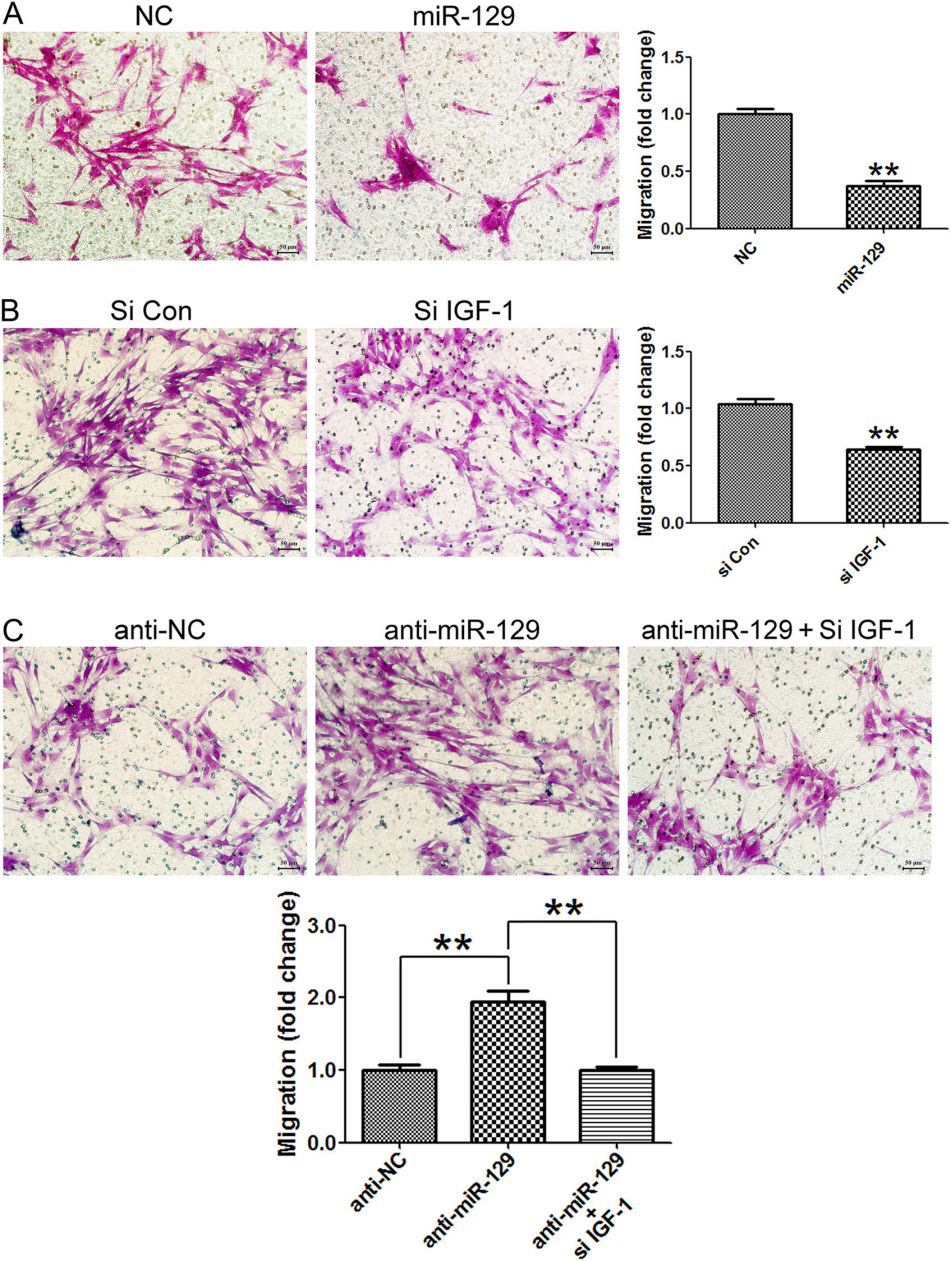
miR-129/IGF-1 axis regulates migration of SCs. **a** The migration rate of SCs transfected with miR-129 was significantly decreased. **b** Knocking down of IGF-1 by siRNA (si IGF-1) significantly inhibited SCs migration as compared with control siRNA. **p < 0.01 versus respective controls. **c** Increase in cell migration of SCs transfected with miR-129 inhibitor (anti-miR-129) was rescued by cotransfection with IGF-1 siRNA (si IGF-1). **p < 0.01 versus respective controls. Scale bar: 50 μm. NC (mimic control), anti-NC (inhibitor control)

The result of migration assay showed that SCs transfected with miR-129 mimic or IGF-1 siRNA induced a significant decrease in cell migration rate compared to SCs transfected with non-targeting negative controls. It suggested that miR-129 could also suppress SCs migration by down-regulating IGF-1 (Fig. 6a, b). In contrast, SCs transfected with miR-129 inhibitor induced a significant increase in cell migration rate compared to SCs transfected with non-targeting negative controls. Besides, miR-129 inhibitor-induced increase in SCs migration was significantly abrogated by IGF-1 knockdown (Fig. 6c).

Collectively, the results further demonstrated that miR-129/IGF-1 axis played an important role in the regulation of SCs proliferation and migration.

### Effects of miR-129/IGF-1/IGF-1R pathway on neurite outgrowth in co-culture system of DRG neurons and SCs

The regenerative microenvironment of injured nerve is important for nerve regeneration. These include the concepts of axon-SC outgrowth partnership such as the secretion of local molecules, which may facilitate or inhibit regenerative activity and the role of directional cues secreted by the SCs to guide regenerated axons. The IGF-1R is a receptor tyrosine kinase, which consists of two ligand-binding extracellular a-subunits associated with two transmembrane kinase domain-containing b-subunits that transduces both IGF-I and IGF-II signals^28^.

**Fig. 7:**
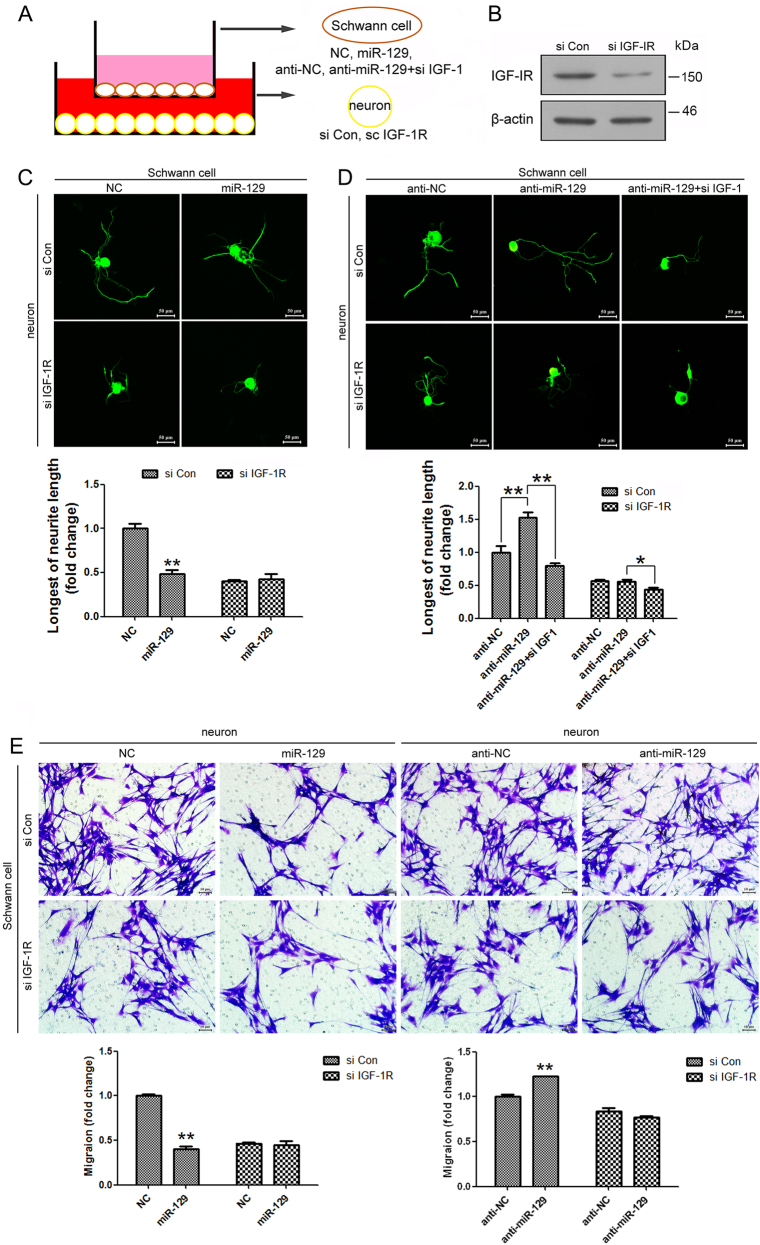
Effects of miR-129/IGF-1/IGF-1R pathway on neurite outgrowth in co-culture system of DRG neurons and SCs. **a** Schematic diagrams of DRG neurons and SCs co-culture system. **b** The protein expression of IGF-1R in DRG neurons transfected with IGF-1R siRNA (si IGF-1R) was significantly decreased as compared to that in neurons transfected with siRNA control (si Con). **c** Immunostaining with anti-β-Tubulin III showing that neurite outgrowth was decreased in co-cultured SCs transfected with miR-129 mimic and DRG neurons transfected with control siRNA, but no change in co-culture with DRG neurons transfected with IGF-1R siRNA. **d** SCs transfected with miR-129 inhibitor increased the neurite outgrowth of DRG neurons, while the effect was abrogated by co-transfection with miR-129 inhibitor plus IGF-1 siRNA in SCs. **e** Migration of SCs was decreased in co-cultures SCs with IGF-1R knocking down and DRG neurons with miR-129 mimic and control, but no change in co-culture with SCs transfected with IGF-1R siRNA. DRG neurons transfected with miR-129 inhibitor increased the migration of SCs, while the effect was not significant in SCs transfected with IGF-1R siRNA. Scale bar: 50 μm. *p < 0.05 and **p < 0.01 versus respective controls. NC (mimic control), anti-NC (inhibitor control)

We applied the DRG neurons and SCs co-culture system to mimic the nerve regenerative microenvironment in vivo and further investigate the regulation of miR-129 on IGF-1 paracrine effects. Primary SCs were transfected with miR-129 mimic and control, miR-129 inhibitor and control, respectively, and then co-cultured with DRG neurons, which have been transfected with IGF-1R siRNA and control previously (Fig. 7a). After IGF-1R knocking down in DRG neurons (Fig. 7b), the significant decrease of neurite outgrowth by miR-129 over-expression in SCs was abolished (Fig. 7c). SCs transfected with miR-129 inhibitor increased the neurite outgrowth of DRG neurons, while the effect was abrogated by co-transfection with miR-129 inhibitor plus IGF-1 siRNA in SCs (Fig. 7d). There was no change in DRG neurons transfected with IGF-1R siRNA, suggesting that the regulation of miR-129/IGF-1 axis was IGF-1R dependent. A parallel experiment that SCs knocking down IGF-1R co-cultured with DRG neurons added with miR-129 mimic and control, miR-129 inhibitor and control (Fig. 7e), revealed that IGF-1 secreted by DRG neurons similarly is important on SC biology.

### In vivo effects of miR-129/IGF-1 axis on axon outgrowth and SCs migration

**Fig. 8:**
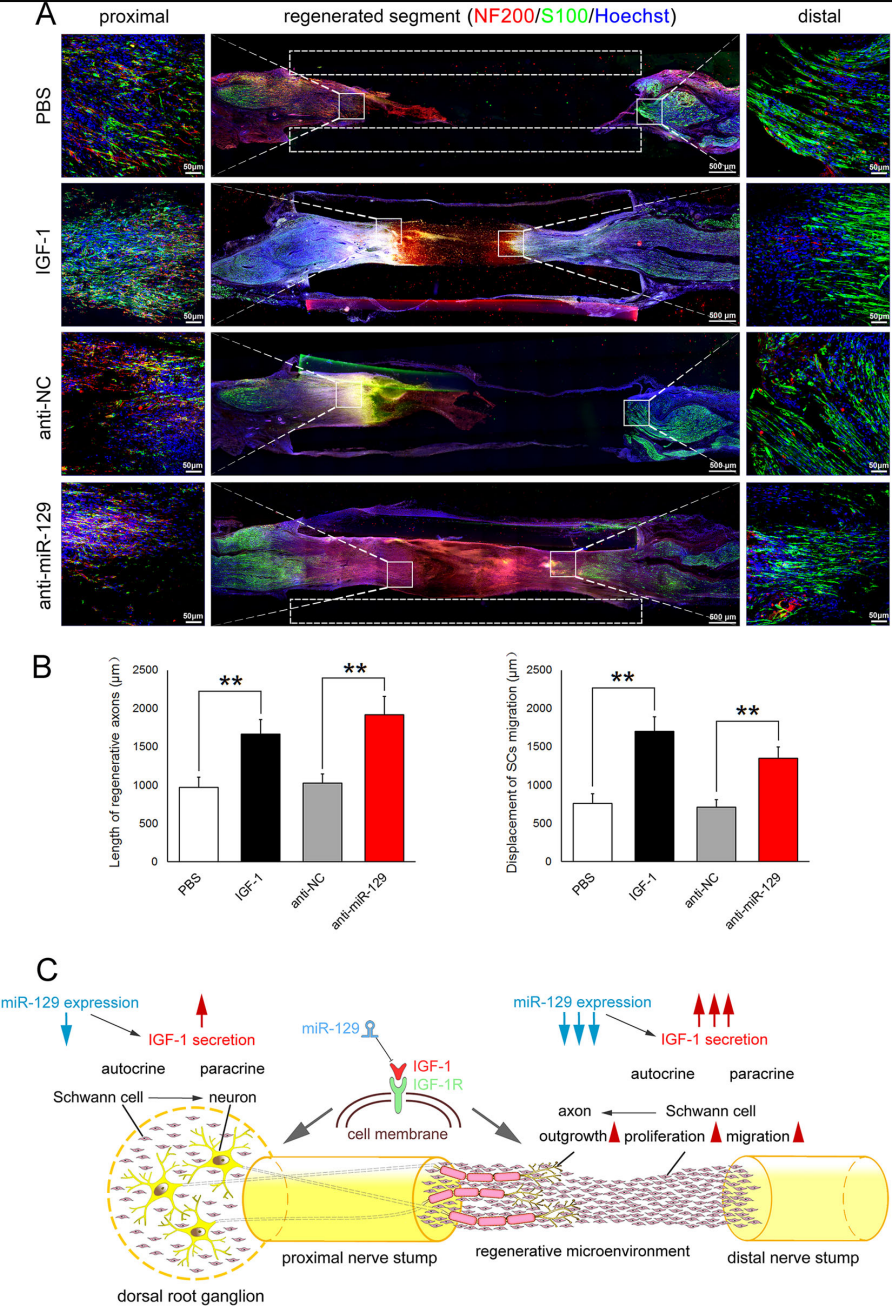
Effects of miR-129/IGF-1/IGF-1R pathway on neurite outgrowth in DRG neurons and SCs in vivo model. **a** Immunostaining with anti-NF200 or anti-S100β showing that both IGF-1 and miR-129 inhibitor promoted axon outgrowth or SCs migration at 10 days after nerve injury, respectively. The length of regenerative axons from the proximal and SCs migration from the distal nerve stump were measured. PBS (PBS vehicle only), IGF-1 (IGF-1 protein, 100 μg/ml), anti-NC (inhibitor control), anti-miR-129 (miR-129 inhibitor). Scale bar: 500 μm. Also shown are the higher magnifications of boxed areas, in which scale bar: 50 μm. **b** Histograms showing quantitative comparisons of the length of regenerative axons and SCs migration at 10 days after implantation as the above mentioned. **p *<* 0.01 versus respective controls. **c** Schematic diagram showing that miR-129/IGF-1/IGF-1R regulated regenerative microenvironment in the injured sites of sciatic nerve and DRG tissues following PNI

Adult rat model with sciatic nerve transection were applied to determine in vivo effects of miR-129/IGF-1 on cell behaviors of neural cells during sciatic nerve regeneration. Following nerve injury, a silicone tube was implanted into the sciatic nerve gap, and miR-129 inhibitor (antagomir) was injected into the silicone tube. Immunostaining with anti-NF200 or anti-S100β showing that both IGF-1 and miR-129 inhibitor significantly promoted axon outgrowth and SCs migration at 10 days after nerve injury, respectively (Fig. 8a, b), which demonstrated the promoting effects of miR-129 inhibitor on peripheral nerve regeneration in vivo.

## Discussion

Growing interests of PNI have fueled exploration of therapeutic approaches that promote spontaneous nerve regeneration. The IGF system is an appealing target for the treatment of PNI based on its potent effects on neuronal development, physiology, and survival. Moreover, miRNAs have the profound potential as therapeutic agents due to their unique properties^29^. The function of miRNAs can be definitely influenced by miRNA agomirs or antagomirs that cause effective and precise upregulation or downregulation^30^. miRNA is easier to deliver into the target cells and less likely to induce high immune response and toxicity than other plasmid DNA-based gene therapy and protein-based drug molecules^31^. This study focused on the possible regulatory role of miR-129 targeting IGF-1 during peripheral nerve regeneration. The expressions of miR-129 in the injured sciatic nerve changed over time following sciatic nerve transection, which suggested that miR-129 might be involved in peripheral nerve regeneration.

SCs are unique glial cells in the PNS and may secrete multiple neurotrophic factors, adhesion molecules, extracellular matrix molecules to form the microenvironment of peripheral nerve regeneration, guiding, and supporting axonal regeneration^32^. During the axonal regeneration, IGF-1 has been shown to play a critical role in SCs by promoting their survival, proliferation, maturation, and differentiation to myelinating phenotypes^33–35^. IGF-1 has been also reported to increase the survival and axonal outgrowth of neurons^36–38^. Our work firstly investigated the target regulation of miR-129 in affecting regenerative microenvironment to further regulate the proliferation and migration of primary SCs, and neurite outgrowth of DRG neurons.

Target therapy by miRNAs provides promising beneficial opportunities with several advantages over traditional small-molecule drugs^39^. Although the favorable therapeutic impact of miRNA has been recognized, several challenges should be considered, including limitation of efficient miRNA delivery to specific cell types, tissues, and organs^40, 41^. Since miRNA targets multiple mRNAs via incompletely matching with their 3′UTRs, miRNAs can also induce the off-target silencing^31^. We previously reported applications of miRNAs in treatment of PNI^23, 42, 43^. Briefly, a regenerative microenvironment in vivo was provided by bridging the rat sciatic nerve gap using the silicone tube, which was added with the mixture of Matrigel and miRNA agomir/antagomir. Further progress will be made in miRNA-therapeutics to regulate many aspects of human disease by preclinical and clinical development since the number of miRNAs targeting the IGF-1 signaling pathway increases^29^. To investigate the regulatory mechanisms of IGF-1, our data from a dual-luciferase reporter assay confirmed that IGF-1 was an exact target gene of miR-129 through directly binding to IGF-1 3′-UTR. Moreover, miR-129 was shown to inhibit IGF-1 expression due to IGF-1 mRNA degradation. Compared to that at 0 h (control), the differential expression levels of IGF-1 at mRNA and protein were more dramatic in the proximal nerve segment than that in DRGs at 4, 7, and 14 days following sciatic nerve injury. It indicates that SCs at the nerve injury site are the main source of IGF-1 and benefit to the regenerative microenvironment. Similarly, the decrease of miR-129 was less dramatic in DRGs than that in the proximal nerve segment. Collectively, IGF-1 was shown to be negatively regulated by miR-129 at post-transcriptional level. It indicates that the regulation of injury-induced secretion of IGF-1 by miR-129 plays crucial roles in the regenerative microenvironment following PNI. Exogenous addition of IGF-1 could significantly increase the neurite outgrowth of DRG neurons, whereas IGF-1-neutralizing antibody reduced the effect. Overexpression of miR-129 inhibited the neurite outgrowth, suggesting that miR-129/IGF-1 axis could regulate neurite outgrowth directly based on its autocrine effect. miR-129/IGF-1 axis is also important in the pre-lesion experiment and in vivo model, which would provide more clinical values regarding the application of IGF-1 or anti-miR-129 in patients with peripheral neural injury.

Furthermore, IGF-1 knockdown prevented an increase in proliferation and migration of SCs by miR-129 inhibitors, which indicated that IGF-1 knockdown could recapitulate the suppressing effects of miR-129 on SCs phenotypic modulation. This interesting finding provided further evidence that IGF-1 was a functional mediator of miR-129.

Besides the regulatory mechanism we have provided here, our knowledge regarding the regulation of IGF-1 secretion contains a calcium sensor synaptotagmin-10 that regulates activity-dependent IGF-1 secretion in olfactory bulb neurons^44^, and Rab8a regulates IGF-1 secretion in a GDP-bound form dependent manner^17^. Moreover, what are the up-regulators of miR-129 following PNI? Following PNI, hypoxia is usually a consequence of lacking blood supply at the injury site. We previously reported the impact of hypoxia on SC behaviors by miRNAs during peripheral nerve regeneration^43^. In addition, angiotensin II (Ang II) encoded by AGT gene, exerts various pathophysiological effects in vasoconstriction, proinflammatory, growth-promoting, and vascular remodeling events. Ang II increases expression of rat miR-129 in cultured vascular smooth muscle cells^45^. Human IL17A protein may also increase expression of human miR-129 in cultured human dermal fibroblasts^45^.

In summary, IGF-1 was up-regulated both in DRGs and the proximal nerve segment following sciatic nerve injury. Dramatic change of IGF-1 in the proximal nerve segment suggested SCs is the main source of IGF-1 at the nerve injury site, which may further promote axonal regeneration. miR-129 negatively regulated IGF-1 by directly targeting its 3′-UTR. miR-129/IGF-1 axis not only regulated neurite outgrowth of DRG neurons directly, but also modified the regenerative microenvironment by regulating SCs proliferation and migration. Upregulation of IGF-1 secretion in SCs at the injury site by miR-129 could promote axonal regeneration indirectly (Fig. 8c). Overall, the regulation on the nerve regenerative microenvironment of injured peripheral nerve is important for nerve regeneration. Our work provides new insight into miR-129 regulation of peripheral nerve regeneration by robust phenotypic modulation of neural cells and opens a novel therapeutic window for PNI by mediating IGF-1 production, which may provide further experimental basis for translation of the molecular therapy into the clinic.
